# Expanding opportunities in health data: Enhancing research by facilitating linkage of Ontario’s population-level health administrative data with data from patient support programs (PSPs)

**DOI:** 10.23889/ijpds.v9i5.2814

**Published:** 2024-09-18

**Authors:** Keresa Arnold, Lisa Ishiguro, Minnie Ho, Dina Skvirisky, Jeruby Retnakanthan, Jacob Etches, Luke Mondor, Saskin Saskin, Charles Victor

**Affiliations:** 1 ICES

## Objectives

Patient Support Programs (PSPs) provide real-world evidence on short-term outcomes and drug adherence for specialty care. These data are collected directly from patients by pharmaceutical companies. Data linkage is facilitated by a publicly-funded health research institute which houses population-based, individual-level health administrative data for 14 million Ontario residents in Canada since 1992. Linkage provides a more comprehensive and transparent view of patients’ pathways and care.

## Approach

Industry-funded research conducted through the data & analytic service must align with the institute’s mission, vision and values, and demonstrate a clear public benefit. For transparency, and to support broader public benefit and research access, final deliverables and analytic plans are posted on the institute’s website. Privacy impact assessments are conducted to ensure ethics approvals, consent and data sharing agreements are established, prior to data importation. To safeguard privacy, designated data covenantors encrypt and link the PSP data with in-house data holdings. Analyses are performed by senior analytic staff, providing researchers with summary-level reports.

## Results

Since 2016, the institute has worked with organizations to link data for research, including PSP data on thousands of patients, enabling crucial insight into treatment patterns, drug adherence, costs and long-term outcomes.

## Conclusion

Linkage of privately-owned PSP data with administrative health data at the institute, provides opportunities to identify gaps in care and improve quality of research. Data challenges around bias, transparency, completeness, and comprehensiveness are minimized.

## Implications

Results provide decision-makers and healthcare professionals with a trusted and comprehensive understanding of patient care pathways to improve health outcomes.

